# Platelet Concentrates in Alveolar and Periodontal Bone Regeneration: Adjunctive Benefits and Clinical Comparability with Conventional Approaches: A Systematic Review

**DOI:** 10.3390/jcm15103617

**Published:** 2026-05-08

**Authors:** Giuseppina Malcangi, Alessio Danilo Inchingolo, Grazia Marinelli, Lucia Casamassima, Paola Bassi, Paola Nardelli, Danilo Ciccarese, Andrea Palermo, Francesco Inchingolo, Massimo Del Fabbro, Angelo Michele Inchingolo, Gianna Dipalma

**Affiliations:** 1 Department of Interdisciplinary Medicine, University of Bari “Aldo Moro”, 70124 Bari, Italy; giuseppinamalcangi@libero.it (G.M.); alessiodanilo.inchingolo@uniba.it (A.D.I.); graziamarinelli@live.it (G.M.); lucia.casamassima@uniba.it (L.C.); paola.bassi@uniba.it (P.B.); paola.nardelli@uniba.it (P.N.); danilo.ciccarese@uniba.it (D.C.); or angelo.inchingolo@unimi.it (A.M.I.); or gianna.dipalma@unimi.it (G.D.); 2 Department of Experimental Medicine, University of Salento, 73100 Lecce, Italy; 3 Department of Biomedical, Surgical and Dental Sciences, Milan University, 20122 Milan, Italy; massimo.delfabbro@unimi.it; 4 Unit of Maxillo-Facial Surgery and Dentistry, IRCCS Foundation Ca’ Granda Ospedale Maggiore Policlinico, 20122 Milan, Italy

**Keywords:** platelet-rich fibrin, periodontal regeneration, alveolar ridge preservation, intrabony defects, bone grafting, guided tissue regeneration

## Abstract

**Background:** Platelet concentrates (PCs), including platelet-rich fibrin (PRF) and its derivatives, have been increasingly investigated as autologous adjuncts in alveolar and periodontal regenerative procedures. This systematic review aimed to evaluate whether PCs improve clinical, radiographic, and histological outcomes compared with conventional regenerative approaches. **Methods:** A comprehensive search of PubMed, Scopus, and Web of Science identified randomized clinical trials on intrabony and furcation defects, alveolar ridge preservation, alveolar cleft reconstruction, and periodontally accelerated osteogenic orthodontics (PAOO). Sixteen studies met the inclusion criteria. **Results:** Overall, PCs, used alone as membranes or in combination with allograft materials, have been associated with significant clinical and radiographic improvements, with three main patterns of effect emerging across studies: (i) adjunctive benefit in selected outcomes (e.g., ridge width preservation and new bone formation when PRF was combined with graft materials); (ii) non-inferiority or equivalence compared with conventional regenerative approaches, particularly in periodontal intrabony defects; and (iii) lack of consistent superiority, as several studies reported comparable outcomes to standard techniques rather than clear advantages. In cleft reconstruction, PRF used in combination with allografts has shown comparable or non-inferior results to standard graft approaches, potentially reducing morbidity. Despite these favorable trends, evidence has been mixed in terms of platelet preparation protocols, defect types, additional biomaterials, and length of follow-up. The heterogeneity of the included studies and the absence of quantitative synthesis limit the strength of the conclusions. **Conclusions:** Within these limitations, PCs appear to represent a valid complement or alternative to conventional regenerative strategies, primarily as an adjunct rather than a clearly superior approach, and further well-designed randomized clinical trials with standardized protocols and longer follow-up are needed to strengthen clinical recommendations.

## 1. Introduction

Alveolar and periodontal bone defects represent a major clinical challenge across implant dentistry, periodontology, and craniofacial rehabilitation. After tooth extraction, the healing socket undergoes a physiologic remodeling cascade characterized by an early inflammatory phase followed by a coupled sequence of bone resorption and formation [1,2]. In particular, osteoclast-driven resorption of the bundle bone and the thin buccal plate—triggered by the loss of periodontal ligament function and vascular supply—plays a central role in the rapid, site-specific reduction of ridge dimensions [3,4,5,6]. Clinically, this translates into a pronounced horizontal and, to a lesser extent, vertical contraction of the alveolar ridge, often occurring within the first months after extraction and potentially compromising ideal implant positioning and long-term aesthetic and functional outcomes [7].

In parallel, periodontal intrabony and furcation defects associated with periodontitis remain biologically demanding to treat, as the ultimate goal is not merely defect fill but reconstruction of a functional attachment apparatus. Conventional regenerative strategies—including open flap debridement (OFD) combined with grafting materials, GTR with collagen membranes (CM), enamel matrix derivatives, and socket sealing techniques—can improve clinical and radiographic parameters, yet outcomes are influenced by defect morphology, biomaterial stability, and the risk of postoperative complications (e.g., membrane exposure), particularly in no contained defects [8,9,10].

Within this framework, PCs have attracted growing interest as autologous biologic adjuncts capable of supporting both soft-tissue maturation and bone regeneration. Platelet-rich fibrin (PRF) and its derivatives provide a fibrin architecture that can function as a scaffold and reservoir for biologically active mediators, potentially enhancing angiogenesis, cellular migration, and early wound healing [11,12,13,14]. Moreover, protocol modifications—such as low-speed PRF and injectable PRF—have been proposed to modulate cellular content and handling properties, enabling PCs to be used as membranes, mixed with graft materials, or applied as biologic fillers/sealants in a variety of clinical indications [15,16,17,18,19,20,21,22].

Randomized clinical evidence suggests that PCs may offer clinically relevant advantages in selected contexts. In alveolar ridge preservation, the addition of injectable PRF to allogenic grafting has been associated with reduced ridge-width contraction and higher newly formed bone percentages compared with grafting alone, supporting the hypothesis that PCs may positively influence both dimensional stability and tissue maturation during early healing [23,24,25,26,27,28,29,30].

Similarly, in periodontal regenerative surgery, adjunctive L-PRF combined with autogenous grafts has shown added benefit over grafting alone in challenging furcation involvement, while in unfavorable intrabony defects L-PRF combined with autogenous grafting has demonstrated non-inferiority to CM-based protocols for key outcomes such as clinical attachment level (CAL) gain [31].

Recent systematic reviews and meta-analyses have evaluated platelet-rich fibrin and related platelet concentrates in selected regenerative contexts, including periodontal intrabony defects, scaffold-assisted bone regeneration, and secondary alveolar bone grafting. However, the available evidence syntheses have mainly focused on single indications, specific PRF derivatives, or restricted biomaterial combinations, thereby limiting cross-context interpretation and direct clinical comparability across alveolar and periodontal applications. In addition, the published literature remains difficult to interpret because of substantial heterogeneity in platelet concentrate preparation protocols, defect morphology, adjunctive grafting materials, outcome measures, and follow-up duration. Such variability across study design and clinical protocols reduces comparability and complicates the interpretation of results. Previous reviews have also highlighted that the reported effects are often indication-specific and not consistently superior to established regenerative techniques. Taken together, these limitations prevent the formulation of clear and generalizable clinical recommendations [7,32].

Accordingly, what remains unresolved is not only whether platelet concentrates may provide benefit in selected clinical scenarios, but also how robust and clinically generalizable those effects are when compared with conventional regenerative strategies across different alveolar and periodontal indications. In particular, the literature still lacks a sufficiently integrated synthesis focused on randomized clinical evidence that jointly examines periodontal intrabony and furcation defects, alveolar ridge preservation, alveolar cleft reconstruction, and periodontally accelerated osteogenic orthodontics within the same comparative framework [32]. 

**Figure 1:**
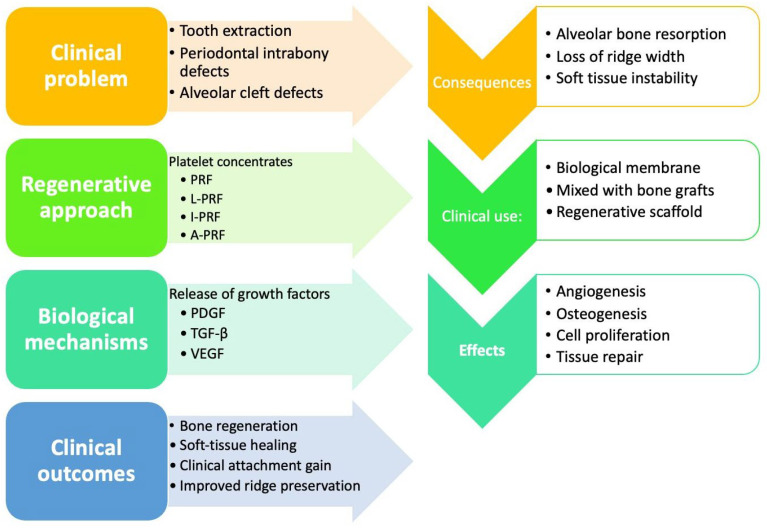
Conceptual diagram illustrating the biological and clinical rationale for the use of PCs in alveolar and periodontal regenerative procedures. PRF and its derivatives release growth factors that may enhance angiogenesis, osteogenesis, and tissue healing, contributing to improved regenerative outcomes.

Therefore, the present systematic review was designed to advance beyond the existing evidence base by providing a structured comparison of randomized clinical trials assessing platelet concentrates across different alveolar and periodontal regenerative procedures, with specific attention to their adjunctive benefits, clinical comparability, and non-inferiority versus conventional regenerative approaches. The aim was not only to summarize favorable outcomes, but also to clarify where the evidence supports a real adjunctive value, where it suggests equivalence only, and where current heterogeneity still prevents definitive clinical recommendations (Figure 1).

## 2. Materials and Methods

### 2.1. Protocol and Registration

The current systematic review followed the PRISMA guidelines (Preferred Reporting Items for Systematic Reviews and Meta-Analyses) (Supplementary Materials) and International Prospective Register of Systematic Review Registry procedures (full ID:1333633).

### 2.2. Search Process

A search of the following databases was conducted in January 2026: PubMed, Web of Science (WOS), and Scopus. These databases were examined for studies published between 1 January 2010 and 30 December 2025 to identify articles from the past 15 years (Table 1).

The search strategy was developed by combining terms relevant to the study’s objectives. In the advanced search queries applied across the databases (with the complete search terms provided in Appendix A), keywords were combined using Boolean operators to capture concepts aligned with the study’s aims (Table 1): (“platelet rich fibrin” OR “PRF” OR “L-PRF” OR “I-PRF” OR “A-PRF”) AND (“periodontal regeneration” OR “intrabony defect” OR “alveolar ridge preservation” OR “socket preservation” OR “alveolar cleft” OR PAOO).

### 2.3. Inclusion and Exclusion Criteria

The reviewers worked in groups to assess all relevant studies that were evaluated. Studies were included if they met the following criteria:

- Open-access articles;
- Studies written in English;
- Studies conducted in vivo or on humans;
- Randomized controlled trials (RCTs);
- Studies published in the last 15 years.

Exclusion criteria comprised review articles, case reports or case series, letters or editorials, in vitro and animal studies.

### 2.4. PICO Question

The PICO format is a framework for structuring clinical research questions in qualitative research. The PICO addressed the question: “In patients undergoing alveolar or periodontal bone regenerative procedures, does the use of PCs improve clinical, radiographic, and histological outcomes compared with conventional regenerative approaches?”.

**Figure 2:**
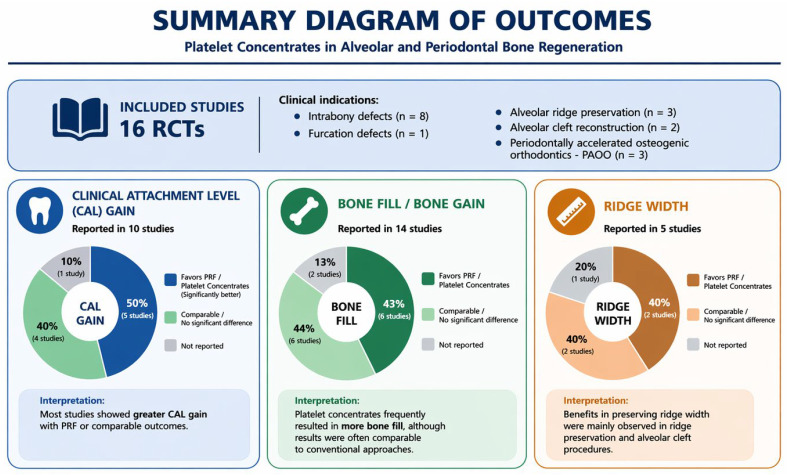
Summary diagram of clinical outcomes (including Clinical Attachment Level (CAL) gain, radiographic bone fill, and alveolar ridge width changes) together with the direction of effect observed across the included studies, illustrating whether each intervention demonstrated a favorable, neutral, or less significant impact compared with control treatments.

The PICO question is developed as follows:

I. Population (P):
  Human patients undergoing alveolar or periodontal bone regenerative procedures, including periodontal intrabony defects; furcation defects; post-extraction alveolar ridge preservation; secondary alveolar cleft grafting; periodontally accelerated PAOO.
II. Intervention (I):
  Use of autologous PCs, including:
  Platelet-Rich Fibrin (PRF);
  Leukocyte-Platelet-Rich Fibrin (L-PRF);
  Injectable Platelet-Rich Fibrin (I-PRF);
  Advanced Platelet-Rich Fibrin (A-PRF);
  PCs may be used as:
  A biological membrane;
  An interpositional layer between graft and soft tissues;
  Mixed with bone grafting biomaterials;
  An adjunct to regenerative surgical procedures;
III. Comparison (C):
  Conventional regenerative approaches performed without PCs, including:
  Bone grafting alone (autograft, allograft, xenograft);
  Bone grafting combined with CM;
  Conventional guided tissue regeneration (GTR);
  Enamel matrix derivative (EMD);
  Open flap debridement (OFD);
  Other standard regenerative techniques without PCs.
IV. Outcome (O):
  **Primary Outcomes (Figure 2):**
  Clinical Attachment Level (CAL);
  Radiographic bone fill/defect bone level gain;
  Alveolar ridge width preservation;
  Percentage of newly formed bone (histological evaluation).
  **Secondary Outcomes:**
  Probing depth (PPD) reduction;
  Gingival recession (GR);
  Marginal bone loss;
  Bone density (CBCT assessment);
  Percentage of residual graft material;
  Postoperative complications;
  Early wound healing.

### 2.5. Data Processing

Four independent reviewers (L.C., P.B., D.C., and P.N.) evaluated the quality of the included studies by examining the selection criteria, the methods used to assess outcomes, and the data analysis procedures. The expanded risk-of-bias tool (Cochrane RoB 2) also offers quality benchmarks related to selection, performance, detection, reporting, and other potential sources of bias. Any disagreements were resolved through discussion or with the involvement of additional researchers (G.M., F.I., A.D.I., and A.M.I.).The reviewers examined all records based on the predefined inclusion and exclusion criteria. A total of 655 eligible articles were imported into Zotero 7.0.31 for management and further analysis.

## 3. Results

### 3.1. Selection and Characteristics of the Study

**Figure 3:**
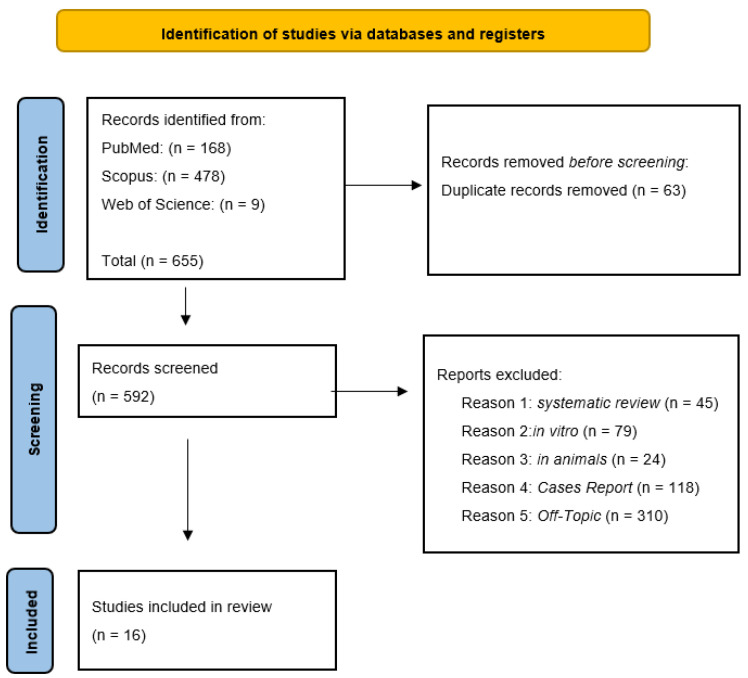
PRISMA flowchart.

This PRISMA (Preferred Reporting Items for Systematic Reviews and Meta-Analyses) diagram (Figure 3) illustrates a rigorous and systematic selection process to ensure that only relevant studies were included in the final review. In total, 655 records were identified through database searching: PubMed (*n* = 168), Scopus (*n* = 478), and Web of Science (*n* = 9). During the identification phase, 63 duplicate records were removed prior to screening, leaving 592 unique records for further evaluation. In the screening phase, the titles and abstracts of these 592 records were assessed according to the predefined inclusion and exclusion criteria. A total of 576 articles were excluded for the following reasons: systematic reviews (*n* = 45); in vitro studies (*n* = 79); animal studies (*n* = 24); cases report (*n* = 118); off-topic or unrelated to the research question (*n* = 310).

After the screening process, 16 studies met all eligibility criteria and were ultimately included in the qualitative synthesis (Table 2).

### 3.2. Quality Assessment and Risk of Bias of Included Articles

The methodological quality of the included manuscripts was assessed by three reviewers (L.C., P.B., and P.N.) using risk-of-bias tools appropriate for the study design. Because all included studies were randomized clinical trials, the Cochrane RoB 2 tool (Risk of Bias 2 for Randomized Trials) was used for their evaluation (Table 3). This instrument examines five key domains: (D1) bias arising from the randomization process, (D2) bias due to deviations from intended interventions, (D3) bias due to missing outcome data, (D4) bias in the measurement of outcomes, and (D5) bias in the selection of the reported results. For each domain, studies were assigned one of four possible judgments—low risk, moderate risk, high risk, or no information—according to the clarity and rigor of the methodological reporting.

In the randomization domain (D1), studies were judged as low risk when adequate sequence generation and allocation concealment (e.g., computer-generated randomization, sealed opaque envelopes) were clearly reported. Studies lacking detailed information on allocation procedures were classified as “some concerns”. Regarding deviations from intended interventions (D2), most studies were considered at low risk, as no relevant protocol deviations were reported and treatments were generally delivered as planned. However, in some cases, the absence of clear blinding procedures led to “some concerns”. For missing outcome data (D3), studies with complete or near-complete follow-up were classified as low risk. When loss to follow-up was reported without sufficient explanation, studies were judged as having “some concerns”. In the outcome measurement domain (D4), studies were considered at low risk when objective and standardized clinical or radiographic measures were used, particularly when outcome assessors were blinded. Conversely, lack of blinding or reliance on subjective measures led to higher risk judgments. Finally, in the selection of reported results (D5), studies were judged as low risk when outcomes were reported consistently with the study aims. In the absence of a published protocol or trial registration, some studies were classified as having “some concerns” due to the potential for selective reporting. Overall, most included studies were judged to have low to moderate risk of bias. Moderate concerns were primarily related to insufficient reporting of randomization procedures and lack of blinding, whereas only a limited number of studies presented a high risk of bias in specific domains. These methodological limitations should be considered when interpreting the results of the present review. Importantly, the presence of mostly low-to-moderate risk of bias across the included studies supports a reasonable level of confidence in the observed clinical trends; however, the remaining methodological shortcomings may still reduce the overall strength and reliability of the evidence, particularly for outcomes related to subjective assessment and selective reporting. Across the included articles, most trials showed low to moderate risk of bias in the domains related to deviations from intended interventions and completeness of outcome data, reflecting generally robust clinical execution and adequate follow-up. Only one study presented a high risk of bias [39], whereas all remaining trials showed low to moderate concerns across the evaluated domains. Conversely, several studies exhibited moderate concerns regarding the randomization process, primarily due to insufficient detail on sequence generation or allocation concealment, and moderate to high concerns in outcome measurement and selective reporting, often linked to lack of blinding or incomplete disclosure of prespecified results. When integrating the domain-specific assessments, the overall risk of bias for the majority of the studies was judged as moderate, indicating acceptable methodological quality but also highlighting areas where reporting transparency and trial design could be strengthened.

### 3.3. Structured Synthesis of Results and Analysis of Heterogeneity

To address heterogeneity, a structured comparison of included studies was performed. Studies were grouped according to clinical indication (intrabony defects, ridge preservation, alveolar cleft reconstruction, and PAOO) and type of intervention (PRF alone vs. PRF combined with grafting materials).

Across intrabony defects, most studies reported improved clinical and radiographic outcomes with PRF, although several trials demonstrated results comparable to conventional regenerative approaches. In ridge preservation and alveolar cleft reconstruction, PRF showed either beneficial or non-inferior outcomes, while in PAOO procedures, PRF appeared to enhance healing and treatment efficiency.

This structured synthesis highlights that PRF may provide benefits in selected clinical scenarios, although results remain heterogeneous and context dependent (Table 4).

To improve interpretability and reduce heterogeneity-related bias, the included studies were grouped according to clinical indication. For each category, results were synthesized by highlighting whether platelet concentrates (PCs) demonstrated superiority, non-inferiority, or no clear benefit compared with conventional regenerative approaches.

#### 3.3.1. Intrabony Defects

The majority of included studies focused on periodontal intrabony defects. When used as an adjunct to grafting materials (e.g., bioactive glass, autogenous bone grafts, or DBBM), PRF was generally associated with superior clinical and radiographic outcomes, including greater clinical attachment level (CAL) gain and bone fill. However, when compared with established regenerative approaches such as collagen membranes combined with grafts, PRF-based protocols consistently demonstrated non-inferiority, with comparable CAL gain and defect fill. In contrast, in more complex or non-contained defects, several studies reported no clear benefit, with outcomes similar to conventional treatments without statistically significant differences. Overall, these findings indicate that PRF is most effective as an adjunct in favorable defect morphologies, while its advantage over standard regenerative approaches is less evident in more challenging clinical scenarios.

#### 3.3.2. Ridge Preservation

In post-extraction ridge preservation procedures, PRF—particularly in its injectable form (I-PRF)—was associated with superior outcomes when combined with grafting materials, including reduced ridge width contraction and increased new bone formation. When used as a standalone approach, PRF demonstrated non-inferiority compared with conventional grafting materials such as freeze-dried bone allografts (FDBA), with similar radiographic and clinical outcomes. Nevertheless, some studies reported no clear benefit in terms of hard-tissue dimensional stability, particularly when PRF was compared with optimized grafting and socket sealing techniques. These findings suggest that PRF may enhance early healing and soft-tissue outcomes, while its impact on long-term ridge preservation remains limited.

#### 3.3.3. Alveolar Cleft Reconstruction

Evidence on alveolar cleft reconstruction is limited but shows consistent trends. When PRF was used in combination with autogenous bone grafts, studies reported superior outcomes in terms of increased bone volume. In comparisons between PRF combined with alternative grafting materials (e.g., xenografts) and autogenous bone grafts, results demonstrated non-inferiority, with comparable clinical and radiographic outcomes. However, PRF did not consistently improve bone density or overall bone quality, and therefore several outcomes can be classified as showing no clear benefit compared with standard approaches. Overall, PRF appears to function primarily as a biologic adjunct that may enhance graft performance and reduce donor-site morbidity, rather than as a replacement for conventional grafting strategies.

#### 3.3.4. PAOO (Periodontally Accelerated Osteogenic Orthodontics)

In PAOO procedures, PRF was associated with superior outcomes in terms of early healing and acceleration of orthodontic tooth movement. When compared with conventional grafting materials or surgical techniques, PRF demonstrated non-inferiority in terms of bone regeneration and periodontal outcomes. However, no consistent additional benefit in long-term hard-tissue outcomes was observed, and several studies reported no clear benefit beyond improved early healing dynamics.

These findings suggest that PRF may be particularly useful for enhancing early biological responses during PAOO, rather than significantly altering long-term regenerative outcomes (Table 5).

## 4. Discussion

### 4.1. Infraosseous Defects (Periodontal Intrabony/Non-Contained Defects)

Regenerative therapy for periodontal intrabony defects has been widely investigated with the aim of improving clinical attachment gain, reducing PPD, and promoting radiographic bone fill [49,50]. In recent years, increasing attention has been directed toward the use of PRF and its derivatives as biologically active adjuncts to conventional periodontal surgery. These biomaterials have been frequently combined with bone grafts or membranes to enhance wound healing and stimulate periodontal regeneration [51,52]. Overall, in this clinical indication PRF appears most beneficial as an adjunct to grafting materials, particularly in contained intrabony defects where regenerative potential is higher.

Early clinical evidence supporting the regenerative potential of PRF was reported by Pradeep et al. (2012), who evaluated the treatment of three-wall intrabony defects using open-flap debridement alone or combined with PRF or PRF plus HA [33,53,54,55,56]. Their results showed that both PRF-based approaches led to significantly greater PPD reduction, clinical attachment gain, and radiographic bone fill compared with surgery alone. However, these findings should be interpreted with caution, as defect morphology (e.g., three-wall contained defects) is inherently associated with more favorable regenerative outcomes and may act as a confounding factor [57,58,59,60,61,62,63,64]. In this context, PRF appears more advantageous in well-contained defects, while its benefit in non-contained lesions remains less predictable.

Comparable findings were reported by Bodhare et al. (2019) [35], who investigated the adjunctive use of PRF in combination with BG for the treatment of intrabony defects. In their split-mouth randomized clinical trial, the addition of PRF resulted in significantly greater clinical attachment gain and radiographic bone regeneration compared with the use of BG alone. Nevertheless, the adjunctive use of osteoconductive biomaterials makes it difficult to isolate the specific contribution of PRF, limiting the strength of causal inference [35,65,66,67,68,69,70]. Clinically, this suggests that PRF is most effective when used as part of combination regenerative protocols rather than as a stand-alone therapy.

In addition to regenerative biomaterials, surgical management may also influence periodontal outcomes. Paolantonio et al. (2020) evaluated periodontal outcomes following different surgical approaches for the exposure of impacted maxillary canines [37]. Although both techniques allowed successful orthodontic alignment, the closed-eruption technique demonstrated more favorable periodontal results, including reduced gingival recession and improved attachment levels [37,71,72,73]. These findings underline the importance of surgical technique in maintaining periodontal tissue stability during orthodontic interventions.

More recent studies have explored the regenerative potential of advanced PRF derivatives. Serroni et al. (2022) investigated the adjunctive use of L-PRF combined with ABG in mandibular molar furcation defects [38]. Their randomized clinical trial showed that regenerative treatments significantly outperformed open-flap debridement alone, with the combination of autogenous graft and L-PRF producing the greatest improvements in PPD reduction and horizontal attachment gain [38,74,75,76,77]. However, when compared across studies, these improvements appear to reflect an adjunctive effect rather than a consistent superiority over established regenerative protocols.

Similarly, Abdulrahman et al. (2022) evaluated the use of low-speed PRF in the treatment of periodontal intrabony defects [39]. Although both treatment groups demonstrated significant clinical improvements, the PRF group exhibited greater attachment gain and PPD reduction, suggesting that PRF may enhance periodontal healing when used as an adjunct to surgical debridement [39,78,79,80,81,82,83]. It should be noted that the comparison with OFD alone may overestimate the clinical benefit, as this approach does not represent a regenerative gold standard.

Further evidence was provided by Balice et al. (2024) [44], who conducted a non-inferiority randomized clinical trial comparing ABG combined with L-PRF to grafts covered with CM in unfavorable intrabony defects. Both approaches resulted in significant improvements in clinical and radiographic parameters. Importantly, the study demonstrated non-inferiority rather than superiority, suggesting that PRF-based approaches may represent a valid alternative rather than a clearly superior option. In clinical terms, this indicates that PRF-based membranes may be a valid alternative to CM in selected cases, rather than a universally superior option [44,53,54,84].

Finally, Almoliky et al. (2025) compared low-speed PRF membranes with CM combined with DFDBA in non-contained intrabony defects [47]. Both treatment modalities produced significant improvements in CAL and PPD, while radiographic bone fill increased over time. No significant differences were observed between groups, suggesting that PRF membranes may provide outcomes comparable to traditional CM in regenerative periodontal therapy [47,85,86,87,88,89]. These findings further support the interpretation that PRF may provide outcomes comparable to conventional regenerative techniques. From a clinical applicability perspective, these findings place PRF in the category of comparable regenerative modalities rather than clearly superior interventions in non-contained defects.

Overall, the available evidence suggests that PRF-based regenerative approaches may provide potential benefits in selected clinical scenarios, particularly in the treatment of intrabony defects and when combined with graft materials, although some studies report clinical outcomes comparable to conventional regenerative techniques [90,91,92,93,94]. Clinically, this positions PRF as most advantageous in contained defects with graft association, comparable in standard regenerative protocols, and less predictable in non-contained lesions.

### 4.2. Socket Preservation (Post-Extraction Ridge Preservation)

Alveolar ridge preservation following tooth extraction is a critical procedure aimed at limiting post-extraction bone resorption and maintaining sufficient bone volume for future implant placement. Various regenerative strategies have been proposed to improve the stability of the alveolar ridge, including the use of autologous biomaterials such as PRF as well as different grafting materials and socket sealing techniques [95,96,97,98,99]. In this indication, PRF appears most useful for enhancing soft-tissue preservation rather than dramatically altering hard-tissue dimensional changes.

The regenerative potential of PRF in ridge preservation procedures was investigated by Talebi Ardakan et al. (2024) [48], who evaluated injectable PRF combined with an allogenic bone graft. Their randomized clinical trial demonstrated that the addition of I-PRF significantly reduced horizontal ridge resorption and increased the percentage of newly formed bone compared with grafting alone. Histomorphometric analysis further revealed fewer residual graft particles in the PRF group, suggesting that PRF may enhance graft integration and accelerate bone remodeling [48,100,101,102,103]. However, the relatively short follow-up period and the combined use of grafting materials limit the ability to determine whether these improvements translate into long-term clinically relevant benefits. Clinically, this supports PRF as an adjunct capable of improving early healing and graft integration, although its long-term superiority remains unproven.

Similarly, Azangookhiavi et al. (2024) [43] compared PRF with FDBA in ridge preservation procedures performed prior to implant placement. Although both treatments demonstrated comparable marginal bone stability and similar bleeding indices, the PRF group showed significantly less gingival recession, highlighting the potential advantages of PRF in improving soft-tissue preservation around future implant sites [43,53,54,104,105]. This suggests that PRF may offer advantages in soft-tissue outcomes, although its overall superiority over established grafting approaches remains uncertain. These findings suggest that PRF is particularly advantageous for soft-tissue outcomes, while remaining comparable to conventional grafting materials for hard-tissue preservation.

In addition to grafting materials, socket sealing methods may also influence ridge preservation outcomes. Camacho-Alonso et al. (2024) [45] compared cyanoacrylate adhesive with CM as sealing materials following ridge preservation procedures in a large randomized clinical trial. Their results showed that the cyanoacrylate group experienced significantly less buccolingual bone width reduction, better CAL, and reduced marginal bone loss compared with the CM group. These findings suggest that socket sealing strategies may play an important role in maintaining alveolar ridge dimensions after tooth extraction [45,106,107]. These findings highlight that surgical technique and defect management may represent important confounding variables, potentially influencing outcomes independently of PRF use.

Taken together, these studies indicate that the combination of biologically active materials and appropriate surgical techniques may contribute to improved hard- and soft-tissue healing during ridge preservation procedures. Nevertheless, the independent contribution of PRF remains difficult to quantify. From a clinical standpoint, PRF in socket preservation should be considered a supportive adjunct rather than a primary determinant of ridge preservation success.

### 4.3. Alveolar Cleft Reconstruction

Reconstruction of alveolar clefts is a complex surgical procedure that aims to restore alveolar continuity, facilitate tooth eruption, and improve maxillary stability. Although ABGs remain the gold standard, increasing attention has been directed toward regenerative strategies that may enhance bone healing while reducing donor-site morbidity. In this indication, PRF appears most beneficial as a biologic enhancer of bone formation and as a strategy to reduce donor-site morbidity rather than as a replacement for autogenous grafting.

One of the earlier studies investigating the role of PRF in cleft reconstruction was conducted by Sawky et al. (2016) [34]. Their randomized clinical trial evaluated the addition of PRF to autogenous iliac crest bone grafts in unilateral alveolar cleft repair. The authors reported a significantly higher volume of newly formed bone in the PRF group compared with autogenous grafting alone. However, bone density values were slightly lower in the PRF group and the difference was not statistically significant, suggesting that PRF primarily enhances bone quantity rather than mineral density [34,108,109,110,111,112]. These results suggest a potential effect on bone quantity rather than overall bone quality.

Although conducted in periodontal defects rather than cleft reconstruction, the study by Eid et al. (2024) [46] provided additional evidence regarding the regenerative capacity of injectable PRF combined with DBBM. The authors reported significantly greater clinical attachment gain and radiographic bone fill when PRF was used as an adjunctive material, supporting the broader regenerative potential of PRF-based biomaterials [46,113,114,115,116,117].

Further evidence in cleft patients was provided by Bedeer et al. (2024) [42], who compared xenograft combined with PRF to traditional autogenous iliac bone grafting in children undergoing secondary alveolar bone grafting. Their randomized clinical study demonstrated that xenograft-PRF achieved bone regeneration comparable to autogenous grafts while avoiding donor-site morbidity and reducing surgical time and hospitalization. These findings suggest that PRF may enable the use of alternative graft materials without compromising clinical outcomes [42,118,119,120,121,122]. Importantly, this supports the role of PRF in reducing donor-site morbidity rather than demonstrating clear superiority in regenerative outcomes.

Overall, current evidence indicates that PRF may enhance bone formation and healing in alveolar cleft reconstruction while potentially reducing the need for autogenous bone harvesting. However, the limited number of studies and heterogeneity in protocols reduce the strength of clinical recommendations. Clinically, evidence is still insufficient to recommend PRF as a standalone or replacement strategy, and its role remains adjunctive.

### 4.4. PAOO as Assisted Regenerative Therapy (Periodontally Accelerated Osteogenic Orthodontics)

PAOO is a surgically assisted orthodontic technique designed to accelerate tooth movement through corticotomy-induced regional acceleratory phenomena and enhanced bone remodeling [116,123,124]. In recent years, regenerative biomaterials and surgical modifications have also been investigated to improve PAOO outcomes, particularly with the aim of enhancing alveolar bone support, graft stability, and periodontal conditions during orthodontic treatment [125,126,127,128]. In this field, PRF appears most advantageous for accelerating early healing and improving soft-tissue response rather than producing consistent additional hard-tissue gains.

Chandra et al. (2019) [36] evaluated the adjunctive use of rhBMP-2 during corticotomy-assisted orthodontic treatment. Their randomized clinical trial demonstrated that the addition of rhBMP-2 significantly increased bone density and reduced orthodontic treatment duration compared with corticotomy alone. Importantly, the use of this biomaterial did not negatively affect postoperative healing or patient-reported discomfort [36,114,117,129].

Another study conducted by Aniruddth Yashwant et al. (2022) compared PRFwith demineralized bone xenograft in PAOO procedures using a split-mouth design [40]. The authors reported that PRF-treated sites exhibited faster orthodontic space closure and improved early soft-tissue healing compared with xenograft-treated sites, suggesting that the biological properties of PRF may enhance the regional acceleratory phenomenon and facilitate orthodontic tooth movement [40,130,131,132,133,134,135].

Finally, Ma et al. (2022) [41] investigated two different surgical coverage techniques during PAOO procedures, comparing periosteum coverage with CM coverage in patients with alveolar bone defects. Both techniques resulted in significant alveolar bone augmentation and stable periodontal outcomes over a one-year follow-up period. However, periosteum coverage demonstrated greater vertical bone regeneration, highlighting the potential influence of surgical design and graft stabilization on regenerative outcomes in PAOO therapy [41,55,136,137,138,139]. Overall, the studies included in this discussion highlight the growing interest in biologically active materials and regenerative strategies across dental therapies. While PRF-based approaches are often associated with favorable outcomes, the available evidence more consistently supports a role as an adjunct or alternative rather than a clearly superior regenerative strategy.

Importantly, the interpretation of these findings should consider the moderate risk of bias, the heterogeneity of clinical indications and protocols, and the lack of standardized PRF preparation methods.

Overall, the studies included in this discussion highlight the growing interest in biologically active materials and regenerative strategies across periodontal, implant, and orthodontic therapies. In general, the use of PRF and related biomaterials has been associated with improvements in clinical and radiographic outcomes, particularly in terms of bone regeneration, clinical attachment gain, and soft-tissue healing. In ridge preservation and alveolar reconstruction procedures, these materials appear to contribute to maintaining alveolar bone dimensions and promoting new bone formation, while in surgically assisted orthodontic techniques they may enhance bone remodeling and facilitate tooth movement [140,141,142,143].

Despite the heterogeneity in study design, biomaterials, and clinical indications, the available evidence suggests that biologically active regenerative approaches represent promising adjuncts in contemporary dental therapies. Nevertheless, further well-designed long-term randomized clinical trials are needed to confirm their long-term effectiveness and to establish standardized treatment protocols.

From a clinical applicability perspective, PRF appears most advantageous in contained intrabony defects and as an adjunct to grafting procedures, shows comparable outcomes to conventional approaches in ridge preservation and PAOO, and remains insufficiently supported as a standalone therapy in alveolar cleft reconstruction.

Furthermore, the relatively short follow-up periods limit the assessment of long-term stability and clinical relevance of the reported outcomes.

Despite these limitations, PRF and related biomaterials remain promising tools in regenerative dentistry. However, further well-designed randomized clinical trials with standardized methodologies and longer follow-up are required to establish more robust clinical recommendations Clinically, this reinforces that PRF should be integrated as an adjunct rather than adopted as a replacement technique.

## 5. Conclusions

Within the limitations of the available evidence, the studies included in this review suggest that biologically active regenerative materials—particularly PRF and its derivatives—may represent valuable adjuncts in several dental surgical procedures. Across periodontal regeneration, ridge preservation, alveolar cleft reconstruction, and surgically assisted orthodontic therapies, these biomaterials were generally associated with improvements in bone regeneration, soft-tissue healing, and clinical outcomes.

PRF-based approaches appear to enhance the biological environment of healing sites through the release of growth factors and the stabilization of graft materials, potentially improving regenerative outcomes when used alone or in combination with bone substitutes. In addition, modified surgical protocols and alternative grafting strategies may reduce treatment morbidity while maintaining comparable clinical results.

Nevertheless, the heterogeneity in study designs, biomaterials, and follow-up periods limits the possibility of drawing definitive clinical recommendations. Future well-designed randomized clinical trials with longer follow-up periods are necessary to confirm the long-term stability of these regenerative approaches and to establish standardized treatment protocols.

Within the limitations of the available evidence, PRF and its derivatives may represent useful adjuncts in alveolar and periodontal regenerative procedures, with potential benefits in selected clinical scenarios.

However, current evidence does not demonstrate consistent superiority over conventional approaches, with several studies reporting comparable outcomes. The observed effects appear to be influenced by multiple confounding factors, including defect characteristics, adjunctive biomaterials, and variability in preparation protocols.

The relatively short follow-up of most studies, together with methodological limitations and moderate risk of bias, further restricts the strength and clinical relevance of the findings.

Therefore, PRF should be considered a complementary option rather than a definitive alternative to established regenerative techniques. Further well-designed randomized clinical trials with standardized methodologies and longer follow-up are required to clarify its clinical indications and effectiveness.

## 6. Limits and Future Limitations

The findings of this systematic review should be interpreted in light of several important limitations. Many of the included studies were characterized by small sample sizes, reducing statistical power and limiting the generalizability of the results.

Substantial heterogeneity was observed across studies in terms of clinical indications, defect types, outcome measures, and adjunctive biomaterials, making direct comparisons difficult and limiting the identification of consistent effects.

In addition, the lack of standardized PRF preparation and application protocols represents a critical source of variability that may influence clinical outcomes.

Most studies reported short- to medium-term follow-up, preventing a reliable assessment of long-term stability and clinical relevance.

Furthermore, several trials presented methodological weaknesses and moderate risk of bias, particularly in relation to randomization procedures, outcome assessment, and reporting, which may contribute to an overestimation of treatment effects.

Finally, the absence of a quantitative synthesis (meta-analysis) limits the ability to provide pooled estimates and strengthens the reliance on qualitative interpretation.

## Figures and Tables

**Table 1:** Indicators for database searches.

Article screening strategy	Keywords: “Platelet-Rich Fibrin; Periodontal Regeneration; Alveolar Ridge Preservation; Intrabony Defects; Bone Grafting; Guided Tissue Regeneration.”
	Boolean Indicators: OR and AND
	Timespan article: 1 January 2010 to 30 December 2025
	Electronic databases: PubMed; Scopus; WOS.

**Table 2:** Analysis of the studies included in the discussion section.

Authors	Type of Study	Number of Ptients	Main Topic	Aim of the Study	Materials and Methods	Conclusions
Pradeep A.R. et al., 2017 [33]	Randomized controlled clinical trial, controlled, double-masked.	62 patients	Regenerative treatment of intrabony periodontal defects	To evaluate the clinical and radiographic effectiveness of PRF alone or combined with HA in 3-wall intrabony defects	Patients with chronic periodontitis treated with OFD alone, OFD + PRF, or OFD + PRF + HA; clinical and radiographic outcomes measured at baseline and 9 months	PRF improves periodontal clinical outcomes and bone fill; addition of HA may enhance regenerative effects and defect fill compared with PRF alone
Shawky H. and Seifeldin S.A., 2016 [34]	Randomized clinical trial, parallel-group design.	24 patients	Effect of PRF in alveolar cleft bone reconstruction	To assess whether PRF improves quantity and quality of bone regeneration in alveolar cleft grafting	Patients with unilateral cleft treated with autogenous iliac crest bone graft ± PRF; CBCT at 6 months evaluated bone volume and density	PRF significantly increases bone volume but does not significantly improve bone density; useful adjunct for enhancing graft quantity
Bodhare G.H. et al., 2019 [35]	Split-mouth randomized controlled clinical trial	20 patients	Effect of PRF combined with bioactive glass (BG) in periodontal intrabony defects	To compare clinical and radiographic outcomes of BG alone versus BG combined with PRF in periodontal regeneration	Paired defects treated with BG alone vs. BG + PRF; clinical measures at 3 and 6 months; CBCT used to evaluate bone fill and defect dimensions	Combination BG+PRF resulted in greater attachment gain and bone fill, indicating enhanced periodontal regeneration compared with BG alone
Chandra R.V. et al., 2019 [36]	Randomized controlled clinical trial, double-blind, parallel-group design.	30 patients	Effect of Recombinant Human Bone Morphogenetic Protein-2 (rhBMP-2) in PAOO on bone density and treatment duration	To evaluate whether rhBMP-2 improves PAOO outcomes in terms of treatment speed, bone quality, healing, and discomfort	Patients randomized to corticotomy alone vs. corticotomy + rhBMP-2; bone density measured radiographically; healing and pain recorded clinically over 6 months	rhBMP-2 reduced treatment time and increased bone density without worsening pain or healing, suggesting potential as a regenerative adjunct in accelerated orthodontics
Paolantonio M. et al., 2020 [37]	Randomized controlled clinical trial (RCT), non-inferiority, parallel-group, prospective	30 patients	Periodontal outcomes of surgical exposure techniques for impacted maxillary canines	To compare periodontal health and stability after open vs. closed canine exposure	Patients treated with open vs. closed surgical exposure followed by orthodontic traction; periodontal indices measured during follow-up	Both techniques effective, but closed-eruption approach showed better periodontal and aesthetic outcomes
Serroni M. et al., 2022 [38]	Randomized controlled clinical trial, parallel-group, prospective	54 patients	Effect of L-PRF adjunct to autogenous bone graft (ABG) in mandibular class II furcation defects	To assess whether adding L-PRF to ABG improves regenerative outcomes compared with graft alone or OFD	Patients randomized into 3 groups: OFD, OFD + ABG, OFD + ABG + L-PRF; clinical and radiographic parameters evaluated at baseline and 6 months	L-PRF combined with ABG significantly improved attachment gain and PPD reduction, providing superior regenerative results
Abdulrahman Y.A. et al., 2022 [39]	Randomized controlled clinical trial, prospective, parallel-group	22 patients	Effect of low-speed PRF adjunct to OFD in intrabony periodontal defects	To determine whether PRF improves clinical attachment gain and defect healing compared with OFD alone	Patients randomized to OFD alone or OFD + PRF; clinical and radiographic parameters assessed up to 9 months	PRF adjunct improved attachment gain and PPD reduction, suggesting it may enhance periodontal surgical outcomes
Aniruddth Yashwant et al., 2022 [40]	Prospective pilot split-mouth clinical study, split-mouth, single-blind (two-arm design)	14 patietns	Comparison of PRF vs. demineralized bone xenograft in PAOO-assisted orthodontic space closure	To compare rate of orthodontic space closure and healing outcomes between PRF and xenograft during PAOO	Split-mouth design; PAOO performed with PRF on one side and xenograft on the other; mini-implant-assisted space closure measured over time; wound healing assessed	PRF promoted faster space closure and better early healing, suggesting it may be an effective biologic alternative to xenografts in PAOO
Zhigui Ma et al., 2022 [41]	Randomized controlled clinical trial, single-blind, parallel-group, prospective.	36 patients	Comparison of periosteum vs. CM coverage in PAOO and effects on alveolar bone regeneration	To determine whether periosteum coverage improves alveolar bone regeneration, periodontal outcomes, and complications compared with traditional PAOO	Adults with Class II/III malocclusion and mandibular defects randomized to periosteum-covered PAOO or collagen-membrane PAOO; CBCT at baseline, 1 week, 12 months; periodontal and complication assessment	Both techniques increased bone volume, but periosteum coverage produced significantly greater vertical bone augmentation and better graft stabilization without worsening periodontal health
Hageer Montaser Bedeer et al., 2024 [42]	Randomized controlled clinical trial (RCT), non-inferiority, prospective, parallel-group, two-arm	36 patients	Comparison of xenograft + PRF vs. autogenous iliac bone graft in secondary alveolar cleft grafting	To determine whether xenograft combined with PRF can provide comparable clinical and radiographic outcomes to ABG	Children with alveolar clefts randomly assigned to autogenous iliac graft or xenograft with PRF; clinical and radiographic evaluation of bone fill, healing, and surgical outcomes	Xenograft + PRF achieved bone regeneration comparable to autograft, reduced operative time and hospital stay, and avoided donor-site morbidity, representing a valid alternative for alveolar cleft reconstruction
Azangookhiavi H. et al., 2024 [43]	Randomized clinical trial, prospective, parallel-group, two-arm	40 patients	Comparison of PRF vs. Freeze-dried bone allograft (FDBA) in alveolar ridge preservation and peri-implant tissue outcomes	To determine whether PRF provides comparable hard- and soft-tissue outcomes to FDBA after ridge preservation and implant rehabilitation	Ridge preservation with PRF or FDBA; implant placement; clinical and radiographic evaluation at 6 and 12 months after loading assessing marginal bone loss, gingival recession, and bleeding	PRF showed similar bone stability and peri-implant health to FDBA but significantly reduced gingival recession, suggesting it may be a valid biologically based alternative for ridge preservation
Balice G. et al., 2024 [44]	Randomized controlled non-inferiority clinical trial (parallel-arm, masked)	64 patients	Treatment of unfavorable intrabony defects using regenerative approaches	To demonstrate non-inferiority of ABG + L-PRF compared to ABG + CM in terms of CAL gain after 12 months	Patients with stage III–IV periodontitis randomly assigned to: (1) ABG + L-PRF or (2) ABG +CM. Clinical (CAL, PPD, GR) and Defect Bone Level measurements at baseline and 12 months. ANCOVA analysis with predefined non-inferiority margin (0.5 mm).	L-PRF + ABG was non-inferior to CM + ABG for CAL gain, showed greater Defect Bone Level gain and lower GR, with slightly higher PPD. Both treatments were effective for unfavorable IBDs.
Camacho-Alonso F. et al., 2024 [45]	Randomized controlled clinical trial, prospective, parallel-group, two-arm.	140 patients	Adjunctive use of Plasma Rich in Growth Factors (PRGF in regenerative treatment of intrabony periodontal defects	To evaluate whether PRGF provides additional clinical and radiographic benefits when combined with Deproteinized Bovine Bone Mineral (DBBM) and CM	Patients with stage III–IV periodontitis randomly assigned to: (1) DBBM + PRGF + CM(test) or (2) DBBM + CM (control). Clinical (CAL, PPD, GR) and radiographic outcomes assessed at baseline and 12 months.	PRGF did not significantly improve CAL gain or radiographic bone fill compared to conventional regenerative therapy. Both treatments were effective and safe.
Eid M.K. et al., 2024 [46]	Randomized controlled clinical trial, prospective, parallel-group, three-arm.	40 patients	Adjunctive use of I-PRF in regenerative treatment of intrabony defects	To evaluate whether adding i-PRF to DBBM improves clinical and radiographic outcomes in intrabony defect treatment	Patients with stage III periodontitis randomly assigned to: Test group (DBBM + i-PRF) or Control group (DBBM alone). Clinical (CAL, PPD, GR) and radiographic assessments at baseline and 6 months.	DBBM + i-PRF resulted in significantly greater CAL gain and radiographic bone fill compared to DBBM alone, indicating improved regenerative outcomes.
Almoliky N. et al., 2025 [47]	Randomized controlled clinical trial, parallel-group, double-blind, prospective, two-arm.	22 patients	Treatment of noncontained intraosseous periodontal defects in stage III periodontitis	To compare clinical and radiographic outcomes of low-speed PRF membrane + DFDBA versus CM + DFDBA in surgical treatment of noncontained intraosseous defects.	Patients randomly assigned to two groups: test (low-speed PRF membrane + DFDBA) and control (CM+ DFDBA). Clinical and radiographic parameters (CAL, PD, Gingival Recession Depth, Full Mouth Bleeding Score, Full Mouth Plaque Score, Radiographic Linear Defect Depth, bone fill) evaluated at baseline and 3, 6, 9, and 12 months.	Both treatments significantly improved clinical and radiographic parameters with no significant intergroup differences; PRF membrane with DFDBA showed outcomes comparable to CM with DFDBA.
Talebi Ardakan et al., 2024 [48]	Double-blinded randomized controlled clinical trial	12 patients	Effectiveness of I-PRF in alveolar ridge preservation after tooth extraction	To compare histological, clinical, and radiographic outcomes of ridge preservation using allograft with and without injectable PRF.	Single-rooted teeth were extracted and randomly assigned to two groups: control (allograft + collagen type I) and test (allograft + collagen type I + I-PRF). CBCT was performed before extraction and after 3 months. Clinical ridge width measurements and biopsies were obtained at implant placement. Histomorphometric analysis evaluated new bone, residual graft, and non-mineralized tissue.	Both treatments were effective for ridge preservation, but allograft + I-PRF showed significantly better bone regeneration and less ridge width reduction compared with allograft alone.

**Table 3:** The table displays the RoB 2 evaluation table for all randomized clinical trials included. Color coding: green = low risk; yellow = moderate risk; red = high risk; blue = no infomation.

Authors and Years	D1	D2	D3	D4	D5	Overall
Pradeep A.R. et al., 2017 [33]	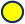	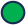	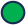	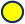	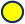	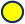
Shawky et al., 2016 [34]	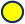	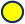	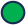	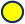	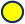	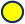
Bodhare et al., 2019 [35]	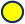	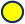	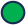	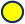	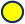	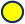
Rampalli Viswa et al., 2019 [36]	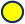	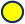	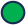	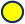	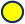	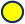
Paolantonio M. et al., 2020 [37]	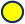	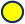	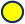	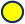	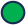	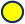
Serroni M. et al., 2022 [38]	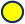	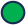	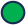	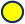	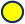	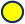
Abdulrahman et al., 2022 [39]	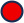	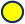	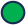	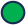	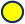	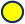
Aniruddth Yashwantet al., 2022 [40]	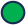	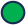	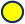	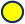	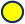	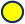
Zhigui Ma et al., 2022 [41]	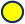	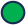	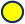	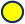	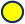	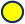
Hageer Montaser Bedeer et al., 2024 [42]	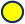	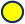	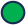	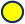	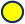	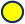
Azangookhiavi, H. et al., 2024 [43]	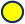	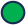	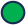	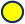	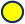	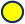
Balice G. et al., 2024 [44]	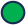	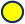	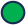	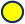	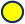	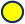
Camacho-Alonso, F. et al., 2024 [45]	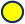	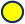	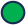	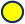	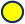	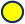
Eid, M.K. et al., 2024 [46]	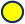	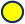	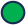	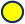	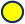	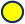
Almoliky N. et al., 2025 [47]	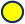	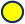	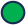	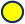	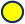	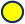
Talebi Ardakani et al., 2024 [48]	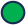	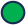	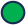	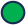	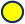	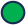
**Domains:**				**Judgement:**		
D1: Bias arising from the randomization process				Moderate risk		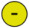
D2: Bias due to deviations from intended interventions				Low risk		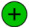
D3: Bias due to missing outcome data				No Information		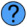
D4: Bias in the measurement of the outcome				High risk		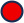
D5: Bias in the selection of the reported result						
Overall: Overall risk of bias						

**Table 4:** The table provides a structured synthesis of the included studies, grouped by clinical indication and intervention type, summarizing main outcomes and the direction of effect (favoring PRF, equivalent, or non-inferior). Note: ↑ indicates an increase; ↓ indicates a decrease.

Clinical Indication	Study	Intervention	Comparison	Main Outcomes	Direction of Effect
***Intrabony defects***	Pradeep 2017 [33]	PRF ± HA	OFD	CAL ↑ PPD ↓ Bone ↑	Favor PRF
***Intrabony defects***	Bodhare 2019 [35]	PRF + BG	BG	CAL ↑ Bone ↑	Favor PRF
***Intrabony defects***	Chandra 2019 [36]	BMP adjunct	Control	Bone density ↑	Favor adjunct
***Intrabony defects***	Paolantonio 2020 [37]	PRF	Control	Similar outcomes	Non-inferior
***Intrabony defects***	Serroni 2022 [38]	PRF + graft	Control	Improved outcomes	Favor PRF
***Intrabony defects***	Abdulrahman 2022 [39]	PRF + OFD	OFD	CAL ↑ PPD ↓	Favor PRF
***Intrabony defects***	Ma 2022 [41]	Membrane vs. periosteum	Control	Bone gain ↑	Equivalent
***Intrabony defects***	Almoliky 2025 [47]	PRF + DFDBA	CM + DFDBA	Similar	Equivalent
***Ridge preservation***	Talebi 2024 [48]	I-PRF + graft	Graft	Bone ↑	Favor PRF
***Ridge preservation***	Azangookhiavi 2024 [43]	PRF	FDBA	Similar	Equivalent
***Ridge preservation***	Camacho-Alonso 2024 [45]	PRGF + DBBM	DBBM	No diff	Equivalent
***Alveolar cleft***	Shawky 2016 [34]	PRF + autograft	Autograft	Bone ↑	Favor PRF
***Alveolar cleft***	Bedeer 2024 [42]	PRF + xenograft	Autograft	Comparable	Non-inferior
***PAOO***	Yashwant 2022 [40]	PRF	Xenograft	Healing ↑	Favor PRF
***PAOO***	Eid 2024 [46]	PRF + graft	Control	Improved	Favor PRF
***Other***	Balice 2024 [44]	PRF + ABG	CM + ABG	Similar	Non-inferior

**Table 5:** Standardized summary of clinical and radiographic outcomes (CAL gain, bone fill, and ridge width) across included studies, with direction of effect. Note: ↑ indicates increase; ≈ indicates similar outcomes.

Study	Clinical Indication	CAL Gain	Bone Fill/Bone Gain	Ridge Width	Effect Direction
Pradeep 2017 [33]	Intrabony defects	↑	↑	–	Favor PRF
Shawky 2016 [34]	Alveolar cleft	–	↑ (volume)	↑	Favor PRF
Bodhare 2019 [35]	Intrabony defects	↑	↑	–	Favor PRF
Chandra 2019 [36]	PAOO	–	↑ (density)	–	Favor adjunct
Paolantonio 2020 [37]	Intrabony defects	≈	≈	–	Non-inferior
Serroni 2022 [38]	Furcation defects	↑	↑	–	Favor PRF
Abdulrahman 2022 [39]	Intrabony defects	↑	↑	–	Favor PRF
Yashwant 2022 [40]	PAOO	–	–	–	Favor PRF (healing)
Ma 2022 [41]	PAOO	–	↑	–	Equivalent
Bedeer 2024 [42]	Alveolar cleft	–	≈	–	Non-inferior
Azangookhiavi 2024 [43]	Ridge preservation	–	≈	≈	Equivalent
Balice 2024 [44]	Intrabony defects	≈	↑	–	Non-inferior
Camacho-Alonso 2024 [45]	Intrabony defects	≈	≈	–	Equivalent
Eid 2024 [46]	Intrabony defects	↑	↑	–	Favor PRF
Almoliky 2025 [47]	Intrabony defects	≈	≈	–	Equivalent
Talebi 2024 [48]	Ridge preservation	–	↑	↑	Favor PRF

## Data Availability

Data are contained within the article.

## Supplementary Materials

The following supporting information can be downloaded at: https://www.mdpi.com/article/10.3390/jcm15103617/s1, PRISMA 2020 Checklist. Reference [144] was cited in Supplementary Materials.

## Abbreviations

The following abbreviations are used in this manuscript:

A-PRF	Advanced Platelet-Rich Fibrin
ABG	Autogenous Bone Graft
BG	Bioactive Glass
CAL	Clinical Attachment Level
CM	Collagen Membrane
DBBM	Deproteinized Bovine Bone Mineral
EMD	Enamel Matrix Derivative
DFDBA	Demineralized Freeze-Dried Bone Allograft
HA	Hydroxyapatite
I-PRF	Injectable Platelet-Rich Fibrin
L-PRF	Leukocyte- and Platelet-Rich Fibrin
OFD	Open Flap Debridement
PAOO	Periodontally Accelerated Osteogenic Orthodontics
PC	Platelet Concentrates
PPD	Probing Depth
PRF	Platelet-Rich Fibrin
PRGF	Plasma Rich in Growth Factors
rhBMP-2	Recombinant Human Bone Morphogenetic Protein-2

## Appendix A

***PubMed query:***

((“platelet rich fibrin”[All Fields] OR PRF[All Fields] OR L-PRF[All Fields] OR I-PRF[All Fields] OR A-PRF[All Fields]) AND (“periodontal regeneration”[All Fields] OR “intrabony defect ”[All Fields] OR “in-trabony defect”[All Fields] OR “alveolar ridge preservation”[All Fields] OR “socket preservation ”[All Fields] OR “alveolar cleft”[All Fields] OR PAOO[All Fields])).

***Scopus query:***

(“platelet rich fibrin” OR PRF OR L-PRF OR I-PRF OR A-PRF) AND (“periodontal regeneration” OR “intrabony defect” OR “in-trabony defect” OR “alveolar ridge preservation” OR “socket preservation” OR “alveolar cleft” OR PAOO).

***Web Of Science query:***

(“platelet rich fibrin”[All Fields] OR PRF[All Fields] OR L-PRF[All Fields] OR I-PRF[All Fields] OR A-PRF[All Fields]) AND (“periodontal regeneration”[All Fields] OR “intrabony defect”[All Fields] OR “intrabony defects”[All Fields] OR “infrabony defect”[All Fields] OR “infrabony defects”[All Fields] OR “intra-bony defect”[All Fields] OR “intra-bony defects”[All Fields] OR “alveolar ridge preservation”[All Fields] OR “socket preservation”[All Fields] OR “alveolar cleft”[All Fields] OR PAOO[All Fields]).
